# Sub-Acute Toxicity Effects of Methanolic Stem Bark Extract of *Entada abyssinica* on Biochemical, Haematological and Histopathological Parameters in Wistar Albino Rats

**DOI:** 10.3389/fphar.2021.740305

**Published:** 2021-09-07

**Authors:** Samuel Baker Obakiro, Ambrose Kiprop, Elizabeth Kigondu, Isaac K’owino, Kenedy Kiyimba, Charles Drago Kato, Yahaya Gavamukulya

**Affiliations:** ^1^ Department of Pharmacology and Therapeutics, Faculty of Health Sciences, Busitema University, Mbale, Uganda; ^2^ Department of Chemistry and Biochemistry, School of Sciences and Aerospace Studies, Moi University, Eldoret, Kenya; ^3^ Africa Centre of Excellence II in Phytochemicals, Textile and Renewable Energy (ACE II PTRE), Moi University, Eldoret, Kenya; ^4^ Centre of Traditional Medicine and Drug Research, Kenya Medical Research Institute, Nairobi, Kenya; ^5^ Department of Pure and Applied Chemistry, Faculty of Science, Masinde-Muliro University, Kakamega, Kenya; ^6^ Department of Pharmacology and Toxicology, School of Pharmacy, Kampala International University, Bushenyi, Uganda; ^7^ Department of Biotechnical and Diagnostic Sciences, College of Veterinary Medicine, Animal Resources and Biosecurity, Makerere University, Kampala, Uganda; ^8^ Department of Biochemistry and Molecular Biology, Faculty of Health Sciences, Busitema University, Mbale, Uganda

**Keywords:** toxicity, fabaceae, traditional medicine, *Entada abyssinica*, biochemical, haematological, histopathalogical, wistar albina rats

## Abstract

**Background:** Whereas the efficacy of *Entada abyssinica* (fabaceae) extracts against various ailments has been scientifically validated, its safety has not been established. This study was undertaken to evaluate the toxicity effects of methanolic stem bark extract of *E. abyssinica* on biochemical, haematological and histological parameters of Wistar albino rats following repeated oral administration.

**Methods:** Wistar albino rats of both sexes were randomized into groups and orally administered daily with determined doses (150, 300 and 600 mg/kg) of *E. abyssinica* methanolic extract using 1% tween 80 in distilled water as a control for 28 days. On the 29th day, all the animals were sacrificed and dissected to collect blood and selected organs. The serum and whole blood were assayed for biochemical and haematological parameters respectively while selected organs were examined for histopathological lesions. Numerical data was analyzed using graph pad prism and expressed as mean ± standard error of mean. The differences between the treatment and control groups were tested for statistical significance using one-way analysis of variance and/or Student’s *t-*test.

**Results:** In repeated daily oral doses (150, 300 and 600 mg/kg), the methanolic stem bark extract of *E. abyssinica* did not cause significant alteration in majority of the biochemical and hematological indices. However, the extract significantly elevated the level of uric acid (all doses), aspartate aminotransferase (300 and 600 mg/kg), low density lipoproteins (150 mg/kg) and mean corpuscular heamoglobin concentration (all doses). On the other hand, the extracts reduced high density lipoproteins (150 and 300 mg/kg), mean corpuscular volume (all doses), haematocrit (150 and 600 mg/kg), mean platelet volume (150 and 600 mg/kg) and procalcitonin (150 mg/kg). In the vital organs, there were no significant lesions observed except at the highest dose (600 mg/kg) where there was mild evidence of lymphocyte infiltration in the liver and focal interstitial nephritis.

**Conclusion:** The methanolic stem bark extract of *E. abyssinica* is relatively safe in Wistar albino rats when repetitively administered orally in small doses for a prolonged period of time. We recommend more chronic toxicity studies and clinical trials on herbal remedies containing this plant to ensure that its use is free of potential toxicity to humans.

## Introduction

Globally, approximately 80% of the world’s population depend on nonconventional therapies for primary health care with herbal products being the most widely utilized (WHO Report on Traditional Medicine, 2019). This is because plants contain abundant secondary metabolites (phytochemicals) with potential pharmacological activity against various diseases. Therefore, the use of herbal medicines in management of several ailments continues to gain momentum in several communities due to their availability, affordability, perceived effectiveness and safety (Omara et al., 2020; Schultz et al., 2020; Tugume et al., 2016). Their use in management of infectious diseases and cancer is even expected to increase due to increasing development of resistance to the available chemotherapeutic agents (Obakiro et al., 2020; Omara et al., 2020)

Toxicity of herbal remedies remains a huge challenge that limits their use despite the general public belief that they are safe and devoid of potential toxicities (WHO Report on Traditional Medicine, 2019). The common toxicities are hepatotoxicity, nephrotoxicity, neurotoxicity, pulmonary toxicity, cardiac toxicity, adult respiratory distress syndrome, seizures, and acute eosinophilic pneumonia (Ko, 2004; Obakiro et al., 2018). The cause of toxicity may be due to presence of inherent toxic secondary metabolites, preparation procedure of the herbal product, variability in active and/or toxic ingredients due to growth conditions and soil chemistry, misidentification of herbs during harvesting, contamination by pathogenic fungi during storage and transport, and adulteration (Anywar et al., 2021; Selamoglu, 2021). Therefore, the World Health Organization (WHO) recommends that herbal remedies undergo rigorous scientific testing for both efficacy and safety so as to protect the public against exposure to poisonous phytochemicals.

*E. abyssinica* (fabaceae) is a deciduous tree with limited branching, spreading flat crown and grows to a height of about 7–10 m tall mainly in East and central Africa. Its stem bark is grey to reddish and the leaves are alternate, pinnate with a round to slightly obtuse apex. The inflorescence has creamy white or fading yellowish, sweet scented flowers. Its fruits are large, flat legumes which splits open to release oval and flat seeds. In several communities, *E. abyssinica* is grown for ornamental and cultural purposes but also harvested from the wild for fibre and wood (Kakudidi, 2004). Traditionally, the stem bark of *E. abyssinica* is harvested and used in preparation of herbal remedies for symptoms of tuberculosis, ulcers, abortion, asthma, cancers, bacterial, and fungal infections (Bunalema et al., 2014; Tibiri et al., 2007; Wagate et al., 2010). Several scientific studies have validated the various pharmacological potential of this plant and reported significant findings. These include; antimycobacterial (Magadula et al., 2012; Mariita et al., 2010), anti-inflammatory (Olajide and Alada, 2001), antibacterial, antifungal, and antioxidant activities (Dzoyem et al., 2017; Tchindaa et al., 2007). Despite the sufficient evidence for its efficacy, there was little scientific evidence to support its safety with regards to its use in herbal medicines. Since there is widespread use of the stem bark of this plant in preparation of herbal remedies for management for tuberculosis and other chronic illnesses, it is necessary to evaluate the toxicity effects of this plant following prolonged repetitive administration. This study was therefore undertaken to evaluate the effect of methanolic stem bark extract of *E. abyssinica* on biochemical, haematological and histological parameters of Wistar albino rats following daily oral administration of the extracts for 28 days.

## Materials and Methods

### Sample Collection, Authentication and Processing

Samples *of E. abyssinica* were collected from their natural habitats in Siaya and Kisumu counties, Western Kenya during the month of January 2020 with the help of a plant taxonomist. The stem barks of the plant were carefully harvested from mature healthy plants with minimal injury to the plant. Leaves, branches and fruits were used to prepare a voucher specimen (OSB/01/2020/001) which was deposited at the University of Eldoret Herbarium, Botany department for correct botanical authentication and reference purposes. The harvested stem barks were packed in sacks and transported to the Chemistry laboratory at Moi University for drying and pulverization. The samples were chopped into small pieces and air-dried under shade at room temperature (25.0 ± 2.0°C) for 4 weeks until a constant mass was obtained. The samples were then pulverized using an electric grinder (NutriBullet^®^ 600 Series), packed and stored in clean labelled paper envelopes at room temperature until extraction.

### Extraction

Methanol of analytical grade was purchased from Merck-Sigma Aldrich and used to extract phytochemicals from the samples. A sample (300 g of powder) was macerated in 1,000 ml of methanol for 3 days with occasional shaking. The mixture was first filtered using cotton wool and then through Whatman’s filter paper No. 1 (pore size 11 µm) to obtain the crude extract. The extract was concentrated to a minimum volume using a rotary evaporator (Hahnvapor HS-2005S) at 40°C and reduced pressure. The concentrated crude extracts were dried in a desiccator over anhydrous copper (II) sulphate to constant weight at room temperature. The concentrated crude extract was stored in clean labeled bottles at 4°C in a refrigerator until further use.

### Experimental Animals and Handling

Mature healthy inbred Wistar albino rats (100–200 g) about 8–10 weeks old were purchased from the animal facility at the College of veterinary medicine, Animal Resources and Biosecurity, Makerere University, Kampala, Uganda. Three animals of the same sex were housed in standard wooden cages (15 × 21 × 29 cm) bedded with wood chips and equipped with continuous flow nipple watering devices. The animals were fed *ad libitum* on standard feeds, allowed free access to water and cage beddings changed every 2 days. The animals were maintained in clean animal facility at 23–27°C, with a 12-h light and darkness cycle. The animals were acclimatized to the housing conditions for 2 weeks prior to commencement of the study and were handled in conformity with guidelines for handling laboratory animals (Kilkenny et al., 2010). Animals which died before the end of the experiment and those sacrificed were pooled in a bio-hazard container and stored at −20°C (for approximately 24 h) before being incinerated. All animals at the end of the experiment were sacrificed under general anesthesia using an overdose of pentobarbitone sodium solution.

### Preparation of Extract Solutions

The concentrated extract (1000 mg) were dissolved in 10 ml of 1% tween 80 in distilled water at room temperature (25.0 ± 2.0°C) to make an extract suspension of 100 mg/ml. The suspension was vortexed (Analog Vortex mixer OHAUS) for 20 min and after digitally shaken (VWR–digital shaker) for 2 h to allow maximum dissolution. The prepared extract solution was then poured in clean labelled flask for administration to the animals. All the extract solutions were freshly prepared every day for use.

### Randomization, Dose Determination and Administration

The OECD 407 guidelines on oral repeated toxicity testing of chemicals in rodents were adopted (Olayode et al., 2019). Wistar Albino rats (24) of both sexes were randomized basing on their body weight into four groups (A–D). Each group consisted of six rats (three males and three females) which were housed in different cages. The administered doses (150, 300, 600 mg/kg) were determined based on the median lethal dose (4183 mg/kg) that was predetermined using the Lorke’s method. Three groups (A, B and C) were orally administered different doses of the extract (150, 300, 600 mg/kg) respectively while group D received 1% tween 80 in distilled water (control) depending on their body weight daily for 28 days. The volume of the extract administered was calculated using the equation below.

$$\mathrm{Volume given to each animal}\left(\mathrm{ml}\right)=\frac{\text{Body weight of the animal }\left(\text{kg}\right)\times \text{Dose }\left(\text{mg/kg}\right)}{\text{Concentration of the extract }\left(\text{mg/mL}\right)}$$ (1)

Observations of any toxic symptoms including death manifested were noted and recorded systematically daily up to the end of the experiment. Body weights of the rats were taken on day 0, 7, 14, 21, and 28. On the 29th day, the surviving rats were sacrificed under general anesthesia using anesthetic ether solution. The rats were dissected to obtain blood and vital organs (liver, kidney, heart, and spleen) for biochemical, hematological and histopathological analyses. The weights of the vital organs were measured using a digital analytical balance.

### Biochemical Analysis

Blood (2 ml) was collected by cardiac puncture from each rat into non-heparinized vacutainers using syringes. Blood was centrifuged at 3,000 rpm for 5 min to obtain serum which was assayed using an automated chemistry analyzer (HumaStar 200) for levels of different biochemical parameters. Test kits for measurement of different parameters were purchased from Sigma-Aldrich and used according to the manufacturer’s instructions. The parameters assayed included creatinine, urea, uric acid, aspartate aminotransferase (AST), alanine aminotransferase (ALT) and alkaline phosphatase (ALP), serum proteins, bilirubin, triglycerides, total cholesterol, Low density lipoproteins (LDL), high density lipoprotein (HDL) and serum electrolytes (Na^+^, K^+^, Ca^2+^, Cl^−^, H^+^).

### Hematological Analysis

Blood (2 ml) was collected by cardiac puncture from each rat into heparinized vacutainers using syringes and analyzed using an analyzer (Sysmex 1000i) for hematological counts of different parameters. These included White blood cell (WBC), neutrophils (NEUT), lymphocytes (LYMP), monocytes (MONO), eosinophil (EO), basophils (BASO), red blood cells (RBC), hematocrit (HCT), hemoglobin (Hb), mean corpuscular volume (MCV), mean corpuscular hemoglobin (MCH), mean corpuscular hemoglobin concentration (MCHC) and platelet count (PCH).

### Histopathological Evaluation

The obtained vital organs (liver, heart, spleen, and kidney) were grossly examined for the observable histomorphological changes and the weight of each organ measured using a digital weighing scale. The isolated organs were fixed in 10% (v/v) buffered formalin labeled bottles for 72 h. After fixation, the tissues were trimmed and loaded in cassettes for processing using an automated tissue processer (Leica 40). They were first dehydrated by placing them in tissue cassettes with graded alcohol concentration (70, 80, 90, and 96%, v/v) and then removed and placed into xylene solution baths to clear off the alcohol. They were then impregnated with molten wax and allowed to dry. The tissues were then sectioned by use of Rotary microtome (at 5 µm thickness), and then stained with hematoxylin and eosin (H & E). Slides were prepared and then examined using a research light microscope connected to computerized camera (Lieca LB_2_–image analyzer). Photomicrographs were captured and then examined for histopathological changes by two independent pathologists who were not aware of the biochemical and hematological data.

### Statistical Analysis

Quantitative data was entered in Microsoft excel version 2013 and its means and standard error of mean calculated. The results were presented as means ± standard error of mean. Statistically significant differences were determined using one-way analysis of variance (ANOVA) and/or Student’s *t-*test followed by Dunnett’s post hoc test using Graph Pad Prism version 5.01 (Graph Pad software, San Diego, California, United States). Differences were considered statistically significant at *p* < 0.05.

### Ethical Approval

The stud was approved and registered by the Scientific and Ethics Research Unit of Kenya Medical Research Institute (KEMR/SERU/CTMDR/CSCP085/4067).

## Results

### Effect of the Extract on Body Weight

All the animals in the treatment and control groups increased in body weight over the 28-day period except for those administered with the highest dose (600 mg/kg). Over the 28-day period, the increase in body weight was only significant (*p* < 0.05) for animals dosed with 300 mg/kg of the extract. There was no statistically significant differences (*p* > 0.05) in the body weight between animals in the treatment and control group (Table 1).

**TABLE 1:** Mean body weights of the rats at different days of the experiment.

Dose (mg/kg)	Mean body weights (g) at different days				
	0	7	14	21	28
150	117.4	130.3 ± 6.62	129.9 ± 5.45	140.2 ± 6.43	149.9 ± 5.5
300	124.4	152.2 ± 4.95^a^	156.8 ± 4.71^a^	155.9 ± 4.43	157.0 ± 4.26^a^
600	108.2	104.6±± 6.7	113.2 ± 4.9	110.5 ± 5.77	115.3 ± 4.68
1% tween 80 in distilled water	110.1	112.4 ± 2.3	117.1 ± 2.56	125.3 ± 3.14	129.9 ± 2.84

Data were expressed as mean ± SEM, *n* = 6.

a significant at p < 0.05.

### Effect of the Extract on Biochemical Parameters

Majority of the biochemical parameters were not significantly altered by administration of the extracts except for serum albumin, AST, Uric acid, LDL and HDL (Table 2). All doses of the extract significantly increased the uric acid levels. The extracts (at 300 and 600 mg/kg) significantly increased (*p* < 0.05) the level of AST but significantly decreased serum albumin (*p* > 0.05). At doses of 150 and 300 mg/kg, the extract significantly decreased the level of HDL. At 150 mg/kg of the extract, the extract significantly increased the level of LDL. All these differences are in comparison with the control (1% tween 80 in distilled water).

**TABLE 2:** Mean levels of biochemical parameters after 28 days at different doses of the extract.

Biochemical parameters	Mean levels of biochemical parameters			
	150 mg/kg	300 mg/kg	600 mg/kg	1% tween 80 in distilled water
Alb (g/dl)	3.52 ± 0.05	3.37 ± 0.05^a^	3.21 ± 0.10^a^	3.56 ± 0.07
Total Protein (g/dl)	6.28 ± 0.13	6.65 ± 0.48	6.617 ± 0.16	6.89 ± 0.23
ALP (U/L)	399.20 ± 69.08	330.50 ± 35.73	447.00 ± 100.10	255.70 ± 43.84
AST (U/L)	145.70 ± 6.47	169.70 ± 16.27^a^	202.3 ± 11.68^a^	119.70 ± 12.93
ALT (U/L)	100.20 ± 7.12	80.33 ± 3.02	84.50 ± 4.5	91.67 ± 6.77
Bilirubin total (mg/dl)	0.16 ± 0.02	0.18 ± 0.02	0.27 ± 0.06	0.26 ± 0.04
Bilirubin direct (mg/dl)	0.25 ± 0.09	0.13 ± 0.01	0.16 ± 0.02	0.06 ± 0.01
Creatinine (mg/dl	0.51 ± 0.04	0.61 ± 0.04	0.60 ± 0.02	0.62 ± 0.01
Urea (mg/dl)	36.12 ± 4.73	32.72 ± 2.95	25.53 ± 1.38	42.85 ± 1.56
Uric acid (mg/dl)	2.55 ± 0.25^a^	2.80 ± 0.27^a^	2.76 ± 0.32^a^	1.66 ± 0.11
Cholesterol (mg/dl)	35.6 ± 58.79	43.00 ± 3.32	46.50 ± 2.63	46.17 ± 3.93
LDL (mg/dl)	11.08 ± 1.68^a^	6.95 ± 0.87	8.45 ± 0.82	4.86 ± 1.11
Trig (mg/dl)	66.85 ± 17.94	72.83 ± 7.30	55.52 ± 10.45	84.00 ± 5.79
HDL (mg/dl)	12.19 ± 4.60^a^	16.43 ± 2.44^a^	31.65 ± 6.67	38.88 ± 4.80

Data were expressed as mean ± SEM, *n* = 6.

a significant at p < 0.05.

### Effect of the Extract on Electrolytes

Extract administration at all doses did not significantly alter the concentration of sodium, potassium, chloride, and calcium ions except at 300 mg/kg where it elevated the potassium ions (Table 3). On the other hand, all doses significantly elevated the hydrogen ion concentration (lowered the pH).

**TABLE 3:** Mean levels of electrolytes after 28 days at different doses of the extract.

Dose (mg/kg)	Mean concentration of electrolytes					
	K+ (mmol/L)	Na+ (mmol/L)	Cl− (mmol/L	ICa2+ (mmol/L)	TCa2+ (mmo/L)	pH
150	4.8 ± 0.11	143.4 ± 0.37	104.2 ± 0.65	0.22 ± 0.07	0.42 ± 0.15	7.018 ± 0.02^a^
300	5.0 ± 0.27**	144 ± 0.47	106.2 ± 0.44	0.51 ± 0.15	0.99 ± 0.29	7.01 ± 0.02^a^
600	4.52 ± 0.05	142.6 ± 0.39	105.5 ± 0.8	0.67 ± 007	1.31 ± 0.14	6.97 ± 0.01^a^
1% tween 80 in distilled water	4.29 ± 0.03	144.3 ± 0.67	104.6 ± 0.65	0.56 ± 0.06	1.06 ± 0.08	7.38 ± 0.17

Data were expressed as mean ± SEM, *n* = 6, Intracellular calcium (ICa^2+^), Total calcium (TCa^2+^).

**Significant elevation of Potassium ions.

a significant at p < 0.05.

### Effect of the Extract on Hematological Parameters

The extract did not have a significant effect (*p* > 0.05) on the white blood cells and its differentials at all doses (Table 4). However, it significantly (*p* < 0.05) altered some red blood differentials (HCT, MCV, MCHC, and RDW-CV) and Platelet differentials (MPV and PCT). At all doses the extract significantly reduced the MCV and HCT while increased the MCHC. MPV was significantly reduced at 150 and 300 mg/kg of extract while PCT at 150 mg/kg only.

**TABLE 4:** Mean levels of hematological parameters after 28 days at different doses of the extract.

Haematological parameters	Mean levels of haematological parameters			
	150 mg/kg	300 mg/kg	600 mg/kg	1% tween 80 in distilled water
WBC (10*3/UL)	8.30 ± 1.62	9.45 ± 1.17	10.52 ± 1.01	12.3 ± 1.37
NEUT (10*3/uL)	1.03 ± 0.16	1.19 ± 0.17	0.795 ± 0.10	1.58 ± 0.28
LYMPH (10*3/uL)	6.37 ± 1.34	7.35 ± 0.96	8.67 ± 0.88	9.73 ± 1.17
MONO (10*3/uL)	0.78 ± 0.18	0.70 ± 0.10	0.90 ± 0.08	0.56 ± 0.11
EO (10*3/uL)	0.11 ± 0.03	0.20 ± 0.05	0.14 ± 0.03	0.19 ± 0.05
BASO (10*3/uL)	0.18 ± 0.03	0.49 ± 0.32	0.15 ± 0.03	0.26 ± 0.05
RBC (10*6/Ul)	7.30 ± 0.43	6.83 ± 1.24	7.25 ± 0.25	7.01 ± 0.31
HGB (g/dL)	13.17 ± 0.69	14.53 ± 0.46	13.28 ± 0.44	13.47 ± 0.22
HCT (%)	45.12 ± 2.09^a^	48.1 ± 1.55	44.93 ± 1.61^a^	52.83 ± 1.32
MCV (FL)	62.05 ± 1.08^a^	58.92 ± 0.29^a^	61.95 ± 0.47^a^	69.92 ± 3.45
MCH (pg)	18.08 ± 0.14	17.82 ± 0.05	18.33 ± 0.18	19.38 ± 0.87
MCHC (g/dl)	29.12 ± 0.28^a^	30.23 ± 0.14^a^	29.60 ± 0.28^a^	27.78 ± 0.22
RDW-SD (fL)	33.25 ± 0.89	33.88 ± 1.02	36.65 ± 2.51	35.23 ± 4.13
RDW-CV (%)	16.65 ± 0.80	18.35 ± 0.81^a^	17.48 ± 1.18	14.68 ± 0.69
PLT (10*3/uL)	544.00 ± 79.23	636.70 ± 64.24	663.30 ± 77.53	774.50 ± 28.42
PDW (fL)	7.83 ± 0.29	7.90 ± 0.14	8.10 ± 0.28	8.57 ± 0.15
MPV (fL)	7.53 ± 018^a^	7.38 ± 0.11^a^	7.57 ± 0.19	8.15 ± 0.11
P-LCR (%)	8.38 ± 1.16	7.28 ± 0.67	8.82 ± 1.25	10.98 ± 0.76
PCT (%)	0.41 ± 0.06^a^	0.47 ± 0.05	0.50 ± 0.05	0.63 ± 0.02

Data were expressed as mean ± SEM, (*n* = 6).

a significant at p < 0.05.

### Effect of the Extract on the Weight of Vital Organs After 28 days

The extract did not significantly alter the weight of the various organs as compared to the control group except for the liver and kidney which were significantly reduced (*p* < 0.05) at 600 mg/kg dose of the extract (Table 5).

**TABLE 5:** Mean weight of vital organs after 28 days at different doses of the extracts.

Dose of extract (mg/kg)	Mean organ weights (g)			
	Kidney	Liver	Heart	Spleen
150	1.03 ± 0.04	5.80 ± 0.30	0.65 ± 0.03	0.90 ± 0.08
300	1.25 ± 0.06	5.80 ± 0.20	0.67 ± 0.03	0.65 ± 0.04
600	0.80 ± 0.03^a^	4.80 ± 0.18^a^	0.55 ± 0.04	0.82 ± 0.07
1% tween 80 in distilled water	1.15 ± 0.06	5.97 ± 0.30	0.63 ± 0.02	0.93 ± 0.09

Data were expressed as mean ± SEM, n = 6.

a p < 0.05.

### Effect of Repeated Doses of the Extract on Histology of the Liver, Kidney, Heart, and Spleen

**FIGURE 1:**
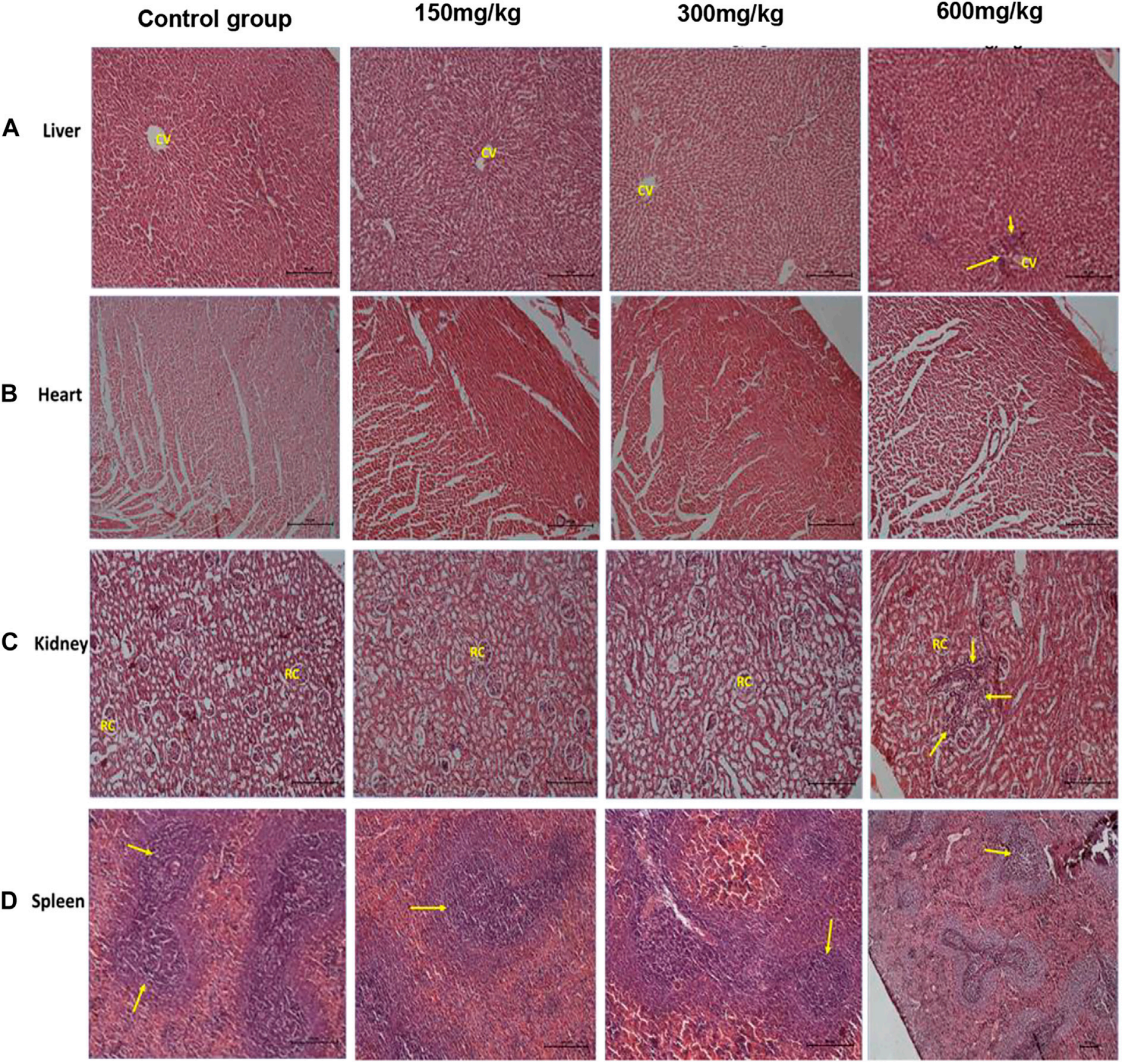
H&E rat organ sections. Panel **(A)** shows normal liver architecture at lower doses with clear central vain, at 600 mg/kg, cellular infiltration is seen (arrow heads). Panel **(B)** shows normal heart section with clear cardiac fibers. Panel **(C)** shows normal kidney architecture at lower doses with clear renal capsules, at 600 mg/kg focal interstitial nephritis is seen (arrow heads). Panel **(D)** shows spleen white pulp hyperplasia due to non-specific immunostimulation at all levels (arrow heads). Each photomicrograph enlargement is 100 µm.

No significant organ lesions were associated with administration of the extract in the heart, liver, kidney and spleen (Figures 1A–D). However, at the 600 mg/kg dose, the extract caused mild to moderate multifocal parenchymal hepatocytes necrosis (Figure 1A), periportal mononuclear inflammatory cell infiltration and focal interstitial nephritis (Figure 1C). In all the treatment and control groups, the spleen exhibited nonspecific immunostimulation as indicated by mild to moderate diffuse lymphoid hyperplasia within the white pulp (Figure 1D). A summary of the organ specific findings is indicated in Table 6.

**TABLE 6:** Summary of organ specific histopathology.

Organ	Histopathology in the different treatment groups			
	1% tween 80 in distilled water	150 mg/kg	300 mg/kg	600 mg/kg
Liver	*No significant lesions*	*No significant lesions*	*No significant lesions*	Mild to moderate multifocal parenchymal hepatocytes necrosis and periportal mononuclear inflammatory cells infiltration
Heart	*No significant lesions*	*No significant lesion*	*No significant lesion*	No significant lesions
Kidney	*No significant lesions*	*No significant lesion*	*Non-significant lesion*	Focal interstitial nephritis
Spleen	*Mild to moderate diffuse lymphoid hyperplasia in the follicles (non-specific immunostimulation)*	*Mild to moderate diffuse lymphoid hyperplasia in the follicles (non-specific immunostimulation)*	*Mild to moderate diffuse follicular lymphoid hyperplasia (non-specific immunostimulation)*	Mild to moderate diffuse follicular lymphoid hyperplasia (non-specific immunostimulation)

## Discussion

In repeated daily oral doses for 28 days, the extract did not significantly increase the body weight over time except for the dose of 300 mg/kg. In the latter group, it is plausible that the extracts were in optimum concentration to stimulate the conversion of nutrients into body tissues. The elevated levels of uric acid in the treatment group indicate a disturbance in the nitrogen metabolism which could probably be due to presence of phytochemicals in the extracts that interact with enzymes or upset the nitrogen metabolism. Additionally, it could as well be due to the extract interfering with renal excretion of uric acid as observed from some histopathologies on the Kidney. Therefore, there is a likely risk of hyperuricemia and gout developing in patients who chronically use herbal remedies that contain this medicinal plant.

The significant increase in AST at higher doses indicated that the extracts could have caused injury to the liver, lungs, heart and kidney. But AST is a non-specific enzyme whose activity/concentration in serum could be due to injury to various vital organs in the body (Akanmu et al., 2020; Mujahid et al., 2017). Histopathological findings revealed that this increase could have been probably due to the mild damage that was observed in the liver and kidney tissues. Changes in LDL and HDL indicated dysregulation of lipid metabolism which could be due to interfering with the process of lipolysis and mobilization of free fatty acids from the peripheral depots (Oluwatoyin et al., 2008; Arunsi et al., 2020; Sowunmi et al., 2020).

The blood indices (white blood cells, red blood cells, platelets and their differentials) serve as an indicator of physiological and pathological status of the body and significant changes imply that the administered chemical is either protective or toxic to the haemopoietic tissue. Findings from our study report non-significant effects on most of the important blood indices by the methanolic extract of *E. abyssinica.* The major functions of WBCs and its differential are to provide immunity and defend the body against invasion by pathogens or toxins. Therefore, the non-significant difference in WBC count and its differentials between the treatment and control groups suggested that the administered doses did not interfere with the differentiation of haemopoietic stem cells into these parameters. The significant effect on red blood cell differentials indicated that the extract affected the process of erythropoiesis probably by the phytochemicals interfering with the secretion and/or activity of erythropoietin (Awe and Banjoko, 2013; Mugisha et al., 2014; Zaruwa et al., 2016). Diminished levels of mean platelet volume and procalcitonin could probably be due to presence of toxic phytochemicals that interfere with the functioning of thrombopoietin or cause inflammation of the bowel (Mwale et al., 2014). The significant lowering of the pH indicated the potential of the extract to cause acidosis probably by stimulating the secretion of hydrogen ions into blood and/or inhibiting their renal excretion.

The low extract dose levels (150 mg/kg, 300 mg/kg) exhibited no significant effect on the histomorphology and gross anatomy of the vital organs. However, at a high dose (600 mg/kg, the extract exhibited a significant decrease in the weight of the liver and kidney in comparison with the control. Histopathological assessment further revealed mild to moderate multifocal parenchymal hepatocytes necrosis and periportal mononuclear inflammatory cells infiltration as well as focal interstitial nephritis. These findings are indicative of infectious or inflammatory lesions although we could not ascertain or propose their actual causes (Singh et al., 2013). These results are in agreement with those reported from methanolic stem bark extract *Entada africana* (a related species) which also did not show significant effect on many biochemical and hematological parameters except a significant increase in the triglycerides and a decrease in Alanine aminotransferase (Tibiri et al., 2007). The later finding indicates the hepatoprotective effects of the extract while the former its risk of hyperlipidemia.

Phytochemical analysis of the methanolic extracts of *E. abyssinica* and *E. africana* revealed presence of alkaloids, tannins, triterpenes, flavonoids, steroid glycosides and coumarins as dominant secondary metabolites (Dzoyem et al., 2017; Yusuf and Abdullahi, 2019). Using ThermoFinnigan LCQ-Duo ion trap mass spectrometer with an ESI source, 28 secondary metabolites were identified from the methanolic stem bark extract of *E. abyssinica.* Majority of these compounds were tannins and gallic acid derivatives. Among the compounds identified was dimethyl caffeoyl galloylglucose, with a retention time of 37.86 min and showed a molecular ion peak at (M–H)− m/z 521 with three daughter ions at 331, 271, and 169. The other compounds were cinnamoyl-O-galloylglucose, p-coumaroyl pyrogalloylgalloylglucose, quercetin galloylglucose, catechin gallate, kaempferol syringyl gallate, gentisic acid dipentoside among others (Sobeh et al., 2020). A new peltogynoid, entadanin and monoglyceride, 1′,26′-bis-[(S)-2,3-dihydroxypropyl]hexacosanedioate along with eight known compounds, were isolated from the stem bark of *E. abyssinica*. The compounds were characterized using 1D and 2D NMR spectra, in combination with high-resolution mass spectrometry, and by comparison with related data from the literature. The other compounds isolated included ursolic acid, quercetin-3-O-β-D-glucosyl (1→4)-α-l-rhamnoside, quercetin-3-O-α-l-rhamnoside (quercitrin), 13,14,15,16-tetranor-3-clerodene-12,18-dioic acid, (8S)-kolavic acid 15-methyl ester, methyl gallate (Melong et al., 2014). These chemical compounds have been reported to possess cytotoxicity, antioxidant and antimicrobial activities (Sobeh et al., 2020; Dzoyem et al., 2017). Eight compounds isolated from this plant had low cytotoxicity with cytotoxic concentrations ranging between 20 and 80 μg/ml (Dzoyem et al., 2017). Therefore these phytochemicals have good pharmacological potential and elicit no-toxicity within possible therapeutic doses (Dzoyem et al., 2017). These *in vitro* findings resonate with our *in vivo* findings which report that the methanolic stem bark of *E. abyssinica* is relatively nontoxic on many biochemical, haematological and histological indices.

## Conclusion

The methanolic stem bark extract of *E. abyssinica* is relatively safe in Wistar albino rats when repetitively administered orally in small doses for a prolonged period of time. We recommend more chronic toxicity studies in animal and clinical trials on herbal remedies containing this plant to ensure that its use is free of potential toxicity to humans.

## Data Availability

The original contributions presented in the study are included in the article/supplementary material, further inquiries can be directed to the corresponding author.
